# Poly-γ-glutamic acid enhanced the drought resistance of maize by improving photosynthesis and affecting the rhizosphere microbial community

**DOI:** 10.1186/s12870-021-03392-w

**Published:** 2022-01-03

**Authors:** Haizhen Ma, Panpan Li, Xingwang Liu, Can Li, Shengkui Zhang, Xiaohan Wang, Xia Tao

**Affiliations:** 1 State Key Laboratory of Biobased Material and Green Papermaking, Qilu University of Technology (Shandong Academy of Sciences), Jinan, 250353 Shandong People’s Republic of China; 2 School of Bioengineering, Qilu University of Technology (Shandong Academy of Sciences), Jinan, 250353 Shandong People’s Republic of China

**Keywords:** γ-PGA, Maize (*Zea mays* L.), Drought resistance, RNAseq, Rhizosphere microbial communities, Plant growth promoting bacteria

## Abstract

**Background:**

Compared with other abiotic stresses, drought stress causes serious crop yield reductions. Poly-γ-glutamic acid (γ-PGA), as an environmentally friendly biomacromolecule, plays an important role in plant growth and regulation.

**Results:**

In this project, the effect of exogenous application of γ-PGA on drought tolerance of maize (*Zea mays*. L) and its mechanism were studied. Drought dramatically inhibited the growth and development of maize, but the exogenous application of γ-PGA significantly increased the dry weight of maize, the contents of ABA, soluble sugar, proline, and chlorophyll, and the photosynthetic rate under severe drought stress. RNA-seq data showed that γ-PGA may enhance drought resistance in maize by affecting the expression of ABA biosynthesis, signal transduction, and photosynthesis-related genes and other stress-responsive genes, which was also confirmed by RT–PCR and promoter motif analysis. In addition, diversity and structure analysis of the rhizosphere soil bacterial community demonstrated that γ-PGA enriched plant growth promoting bacteria such as *Actinobacteria*, *Chloroflexi*, *Firmicutes*, *Alphaproteobacteria* and *Deltaproteobacteria*. Moreover, γ-PGA significantly improved root development, urease activity and the ABA contents of maize rhizospheric soil under drought stress. This study emphasized the possibility of using γ-PGA to improve crop drought resistance and the soil environment under drought conditions and revealed its preliminary mechanism.

**Conclusions:**

Exogenous application of poly-γ-glutamic acid could significantly enhance the drought resistance of maize by improving photosynthesis, and root development and affecting the rhizosphere microbial community.

**Supplementary Information:**

The online version contains supplementary material available at 10.1186/s12870-021-03392-w.

## Background

As one of the major adverse environmental stresses that hinders crop productivity worldwide, the threat of drought stress is increasing due to global climate change [1–4]. The growth and development of plants require sufficient water, and water shortages can be fatal to crops and lead to yield losses. Moreover, drought stress also causes a series of other problems, such as soil erosion, land desertification, ecosystem destruction and so on [5–7]. The shortage of water resources has been considered to be an urgent global environmental issue. Water scarcity has aroused great concern, and the effect of drought stress on plants is receiving increasing attention. To improve the drought resistance of crops, the morphological, physiological, metabolic, molecular and genetic mechanisms of drought resistance of various plants have been systematically studied [7–12]. Conventional breeding, molecular marker-assisted selection, plant transgenic technology, the exogenous application of hormones (such as ABA) or osmoprotectants (such as glycine betaine and proline) and drip irrigation are all used as technical strategies to cope with drought stress [13–21].

Poly-γ-glutamic acid (γ-PGA) is a nontoxic, water-soluble, biodegradable and environmentally friendly biopolymer that is composed of D/L-glutamic acid monomers and is fermented by *Bacillus subtilis* [22, 23]. In line with its different molecular weights, γ-PGA could be used in many fields, such as food, medicine, cosmetics and agriculture [24]. γ-PGA has received increasing attention as an environmentally friendly fertilizer synergist because of its strong water solubility and retention, biodegradability and innocuity [25]. Recent studies have found that γ-PGA plays an important role in plant growth and regulation and can be used as a water retaining agent and soil conditioner to improve crop productivity [26–28]. It has been reported that exogenous application of γ-PGA could significantly enhance the stress resistance of plants [26, 29–31]. Most of the previous studies focused on cold and salt stress in vegetables such as *Brassica napus* and cucumber. For example, it was found that γ-PGA could increase the salt and cold tolerance of *Brassica napus* by activating the crosstalk between H_2_O_2_ and Ca^2+^ signals [32] and enhance the drought resistance of *Brassica napus* by promoting ABA accumulation. However, only a few studies have assessed the effect of γ-PGA on the drought resistance of plants, especially crops. The regulatory mechanism of γ-PGA in the drought resistance of maize remains unclear. Maize is an important crop that is used for grain, feed, energy and industrial raw materials, and it plays an extremely important role in worldwide food security and economic development [33]. Its yield is always severely affected by drought stress [34]. In this study, the effect of γ-PGA on the growth of maize seedlings under drought stress was assessed by adding γ-PGA to the soil. In addition, RNA-seq was performed to study the gene expression of maize leaves after drought stress. The changes of rhizosphere microbial community after exogenous application of γ-PGA were also studied to understand the mechanism by which the exogenous application of γ-PGA to changes the drought resistance of maize.

## Results

### Exogenous application of γ-PGA enhanced the drought resistance of maize

To investigate the effect of the exogenous application of γ-PGA on maize under drought stress, the drought-resistant phenotype of maize treated with different concentrations of γ-PGA (0, 50, 70 and 100 mg/L) was examined (Additional file 1: Fig. S1). We calculated the surviving rate after 7 days of drought, and most of the control maize plants showed severe wilting and could not grow again after rewatering. Only 14.44% of the control maize could resume growth. But the most (82.64–87.5%) of the maize treated with γ-PGA could regenerate maize plants rapidly after rewatering, and there was no significant difference in the survival rate among the three concentrations of γ-PGA treatment. The results showed that the addition of γ-PGA could significantly enhance the drought resistance of maize, even at a lower concentration (50 mg/L). Therefore, the 50 mg/L γ-PGA treatment was used for the subsequent experiments.

**Fig. 1:**
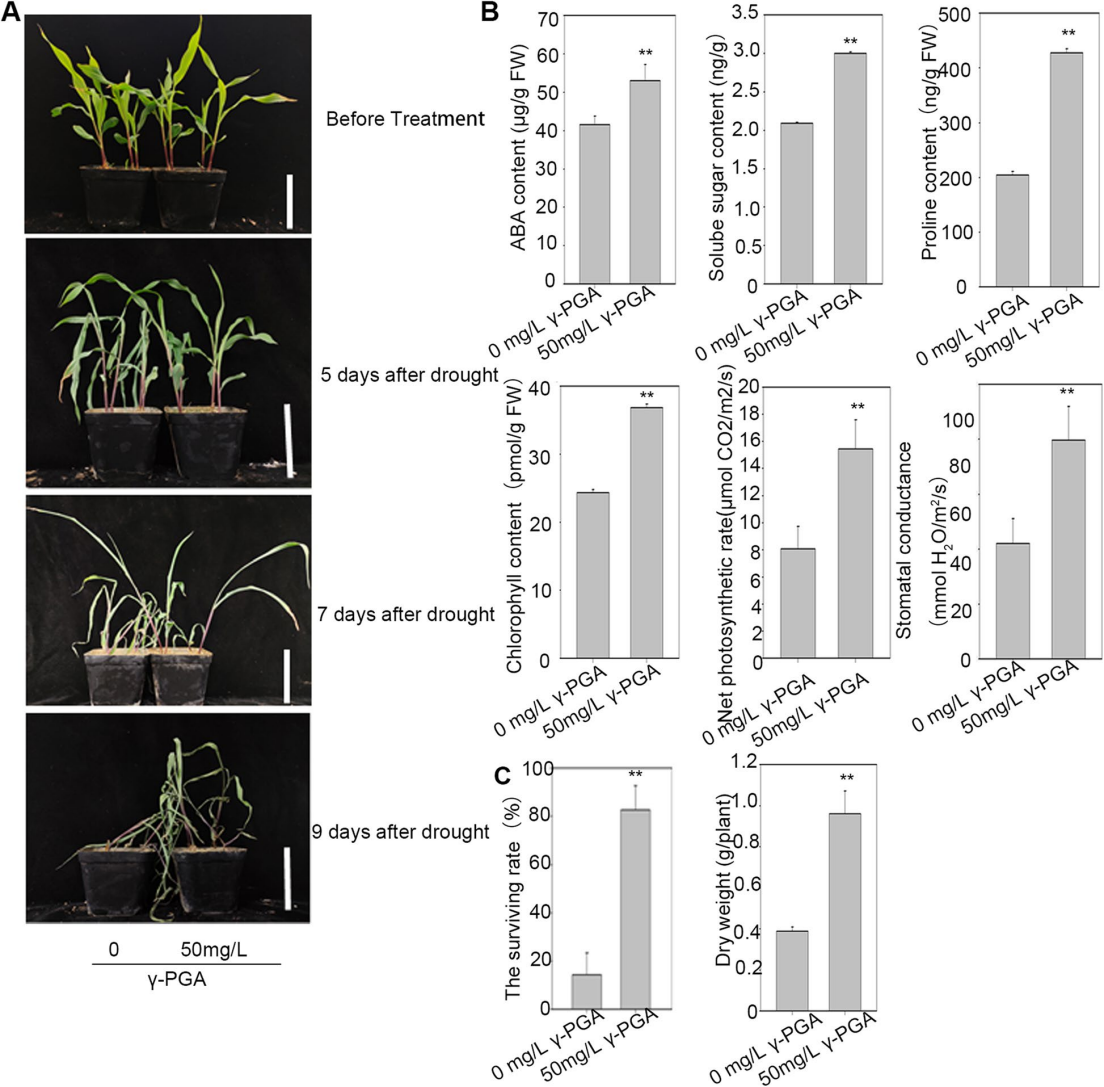
Phenotypes of maize supplemented with 50 mg/L γ-PGA under drought stress, and the determination of related physiological indices. **A** Phenotypes of maize supplemented with 50 mg/L γ-PGA under drought stress. **B** The determination of related physiological indexes (contents of ABA, soluble sugar, proline, and chlorophyll; and the net photosynthetic rate stomatal conductance) after drought for 5 days, Values are means ± sd (*n* = 3 repeats for determination of the contents of ABA, soluble sugar, proline, and chlorophyll, and *n* = 5 repeats for determination of the net photosynthetic rate and stomatal conductance); **C** The dry weight and the surviving rate after drought stress for 7 days. Values are means ± sd (n = 5 repeats). Significant differences are indicated by asterisks (**, *P* ≤ 0.01)

Maize treated with 50 mg/L γ-PGA exhibited a better phenotype after 7 days of drought stress (Fig. 1A). As shown in Fig. 1C, under drought conditions for 7 days, the dry weight of maize treated with γ-PGA (0.96 g) was significantly higher than that of control maize (0.39 g), indicating that γ-PGA could alleviate the inhibition of drought stress on the growth of maize seedlings. The survival rates of the plants were also calculated after 7 days of drought treatment. The results showed that after 7 days of drought treatment, most of the control maize died, only 14% of the plants survived after rewatering, and the maize plants supplemented with γ-PGA were also wilted badly, however, 82.64% of the plants could resume growth after rewatering (Fig. 1C). To analyze the drought resistance of the maize plants, the dry weight, content of ABA, soluble sugar, proline, chlorophyll and photosynthetic parameters of maize seedlings after 5 days of drought treatment were determined. In addition, compared with the control group, the contents of ABA, soluble sugar, proline and chlorophyll in the γ-PGA treatment group were 27.46, 43.61, 108 and 51.5% higher, respectively (Fig. 1B). This indicated that γ-PGA could promote the accumulation of ABA, soluble sugar, proline and chlorophyll in maize under drought stress. The net photosynthetic rate of maize under drought for 5 d were also measured, the results showed the net photosynthetic rate and stomatal conductance of maize with added γ-PGA were significantly higher than that of control maize under drought stress (Fig. 1B).

**Fig. 2:**
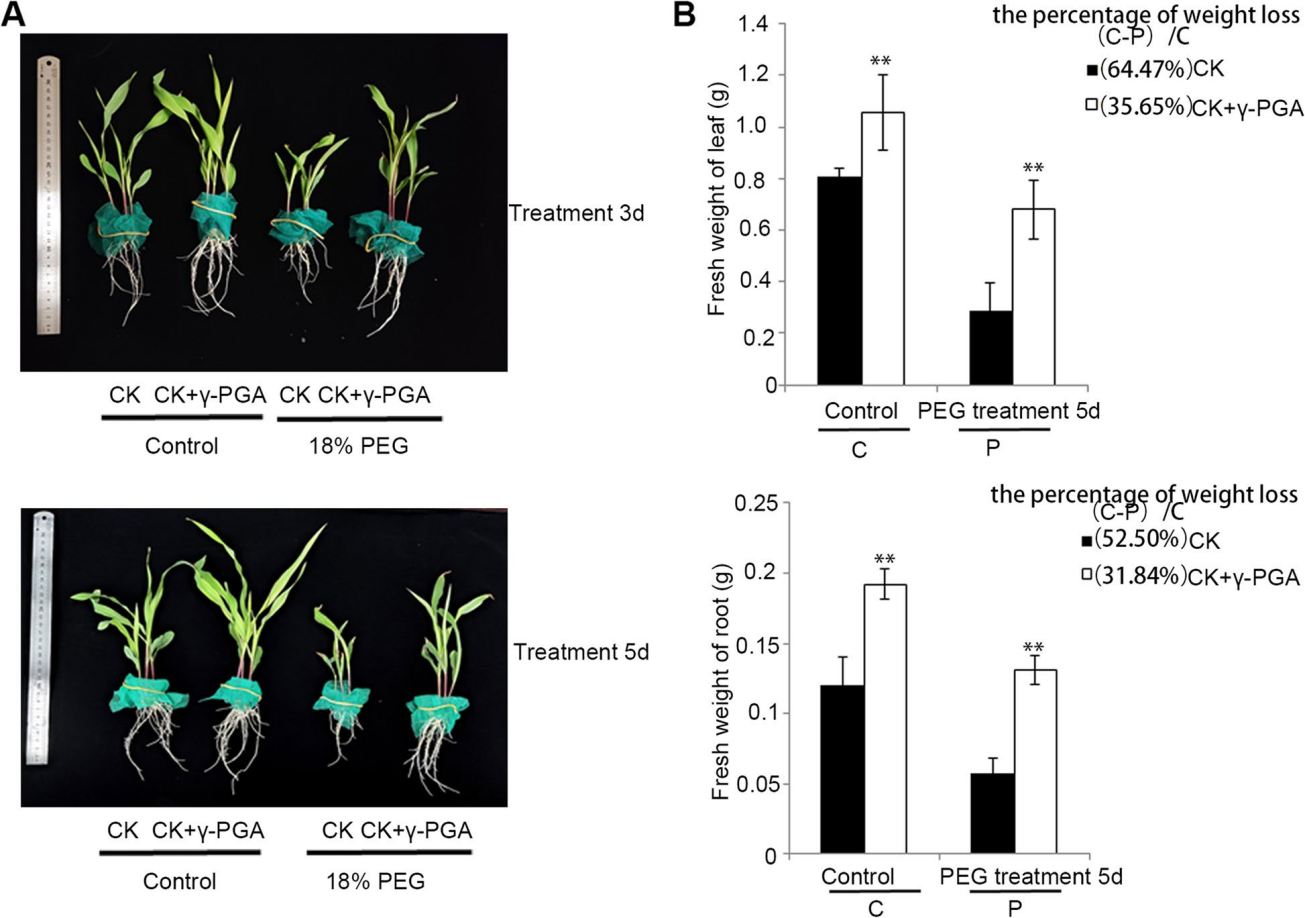
Phenotypes of maize with added γ-PGA under drought stress treatment with 18% PEG6000 solution. **A** Phenotypes of maize with added γ-PGA (10 kDa, 50 mg/L) under drought stress treatment with 18% PEG6000 solution. **B** Fresh weight of the leaves and roots of maize with added γ-PGA and control maize under drought stress treatment for 5 d with 18% PEG6000 solution. Values are means ± sd. Bars represent means ± sd (n = 5 repeats). Significant differences are indicated by asterisks (**, P ≤ 0.01). Bars = 5 cm

To observe the effect of γ-PGA on maize growth under drought stress more directly, a simulated drought experiment with 18% PEG6000 solution was performed. The fresh weight of leaves and roots in the γ-PGA treatment group was higher than those in the control group under both normal and PEG treatment conditions. We also evaluated the weight loss rate of roots and leaves under drought stress after γ-PGA treatment, and the results showed that the reduced fresh weight of maize with γ-PGA treatment (reduced 35.65% in leaves and 31.84% in roots) under PEG treatment was lower than that in the control maize plants (reduced 64.47% in leaves and 52.50% in roots) (Fig. 2). To analyze the drought resistance of the maize plants under the PEG treatment, the solute potential and the relative water content (RWC) of the maize leaves were also determined. As shown in the Additional file 2: Fig. S2A, the maize supplemented γ-PGA exhibited higher osmotic adjustment ability under PEG treatment. When treatment with PEG, the RWC of the maize seedlings showed a sharp decrease, however, the maize treatment with γ-PGA also showed a higher RWC compared the control maize (Additional file 2: Fig. S2B). And the results indicated that the drought resistance of leaves and roots in the γ-PGA + PEG group was significantly higher than those in the control group.

### γ-PGA significantly improved root development, urease activity and ABA contents of maize rhizospheric soil under drought stress

γ-PGA significantly improved the root development under both normal conditions and drought stress (Fig. 2A). Under normal growing conditions, the maize treated with γ-PGA had a better developed root system, and the fresh weight of roots was significantly increased compared with that of the control group. Under PEG simulated drought stress, the root growth of the control group was significantly inhibited, however, the roots of the maize treated with γ-PGA were little affected by drought stress, and the root fresh weight was significantly higher than that of the control group. Since maize rhizospheric soil was in close contact with the roots, the urease activity (closely related to soil nitrogen transformation) and ABA content (closely related to drought resistance) of the maize rhizospheric soil under severe drought stress were also detected. It was observed that the urease activity of rhizospheric soil of γ-PGA treatments increased by 27.74%, while the ABA content of soil under γ-PGA treatments increased by 21.70% (Table 1).

**Table 1:** Effect of γ-PGA on the contents of ABA and Urease activity of maize rhizospheric soil under severe drought stress

Drought	ABA (μg/g DW)	Urease activity (μg NH3-N/g/24 h)
0 mg/L γ-PGA	1.479 ± 0.011	767.583 ± 124.714
50 mg/L γ-PGA	1.800 ± 0.002**	980.524 ± 46.475**

Values are means ± sd (*n* ≥ 3 repeats). Significant differences are indicated by asterisks (**, *P* ≤ 0.01)

### Differentially expressed genes (DEGs) between maize with γ-PGA addition and the control under drought stress

**Fig. 3:**
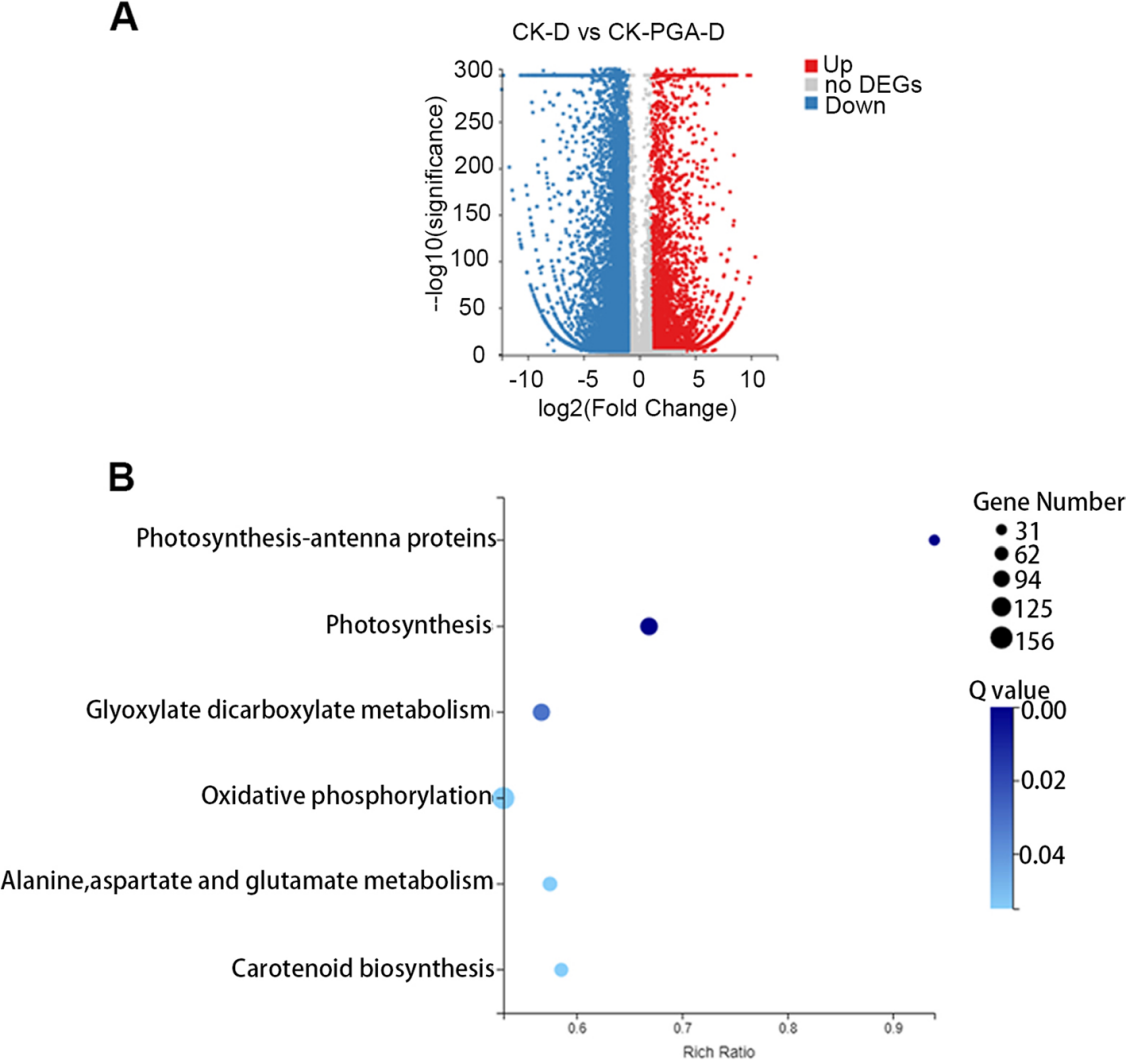
The differentially expressed genes (DEGs) identified by RNA sequence analysis and KEGG enrichment analysis of DEGs. **A** The number of differentially expressed genes (DEGs) identified by RNA sequence analysis. **B** The KEGG enrichment analysis of DEGs, Q value≤0.05

To explain the mechanism of γ-PGA in improving the drought resistance of maize, the leaves of γ-PGA treated plants and control maize plants under drought conditions for 5 d were used for RNA sequencing to identify the DEGs and pathways in response to drought stress. The total raw reads, clean reads, genome mapping ratio, and unique mapping ratio was listed in Additional file 11: Table S1. A total of 16,126 DEGs were identified and the distribution of the DEGs was illustrated in Fig. 3A. These DEGs were subjected to KEGG pathways and Gene Ontology (GO) function enrichment analyses. Based on KEGG pathway analysis, all DEGs were significantly enriched into 6 pathways (Q value≤0.05) namely photosynthesis-antenna proteins (31 DEGs), photosynthesis (105 DEGs), glyosylate dicarboxylate metabolism (102 DEGs), oxidative phosphorylation (156 DEGs), alanine, aspartate and glutamate metabolism (73 DEGs) and carotenoid biosynthesis (65 DEGs) (Fig. 3B). The results of GO annotation function enrichment analysis also showed that GO terms such as photosynthesis and photosystem, response to abiotic stimulus, chlorophyll metabolic process, response to biotic stimulus, electron transport chain and so on were significantly enriched (Additional file 3: Fig. S3A). A more detailed classification of the terms involved in the response to abiotic stimulus showed that these DEGs were mainly related to the response to stress (osmotic stress, salt, heat, cold, reactive oxygen species, and hydrogen peroxide), the response to hormones (ABA, JA, and SA), ABA biosynthetic process, chlorophyll metabolic process, proline biosynthetic process, protein folding, and others (Additional file 3: Fig. S3B).

### γ-PGA improved the drought resistance of maize by affecting the expression of photosynthesis-related genes

**Fig. 4:**
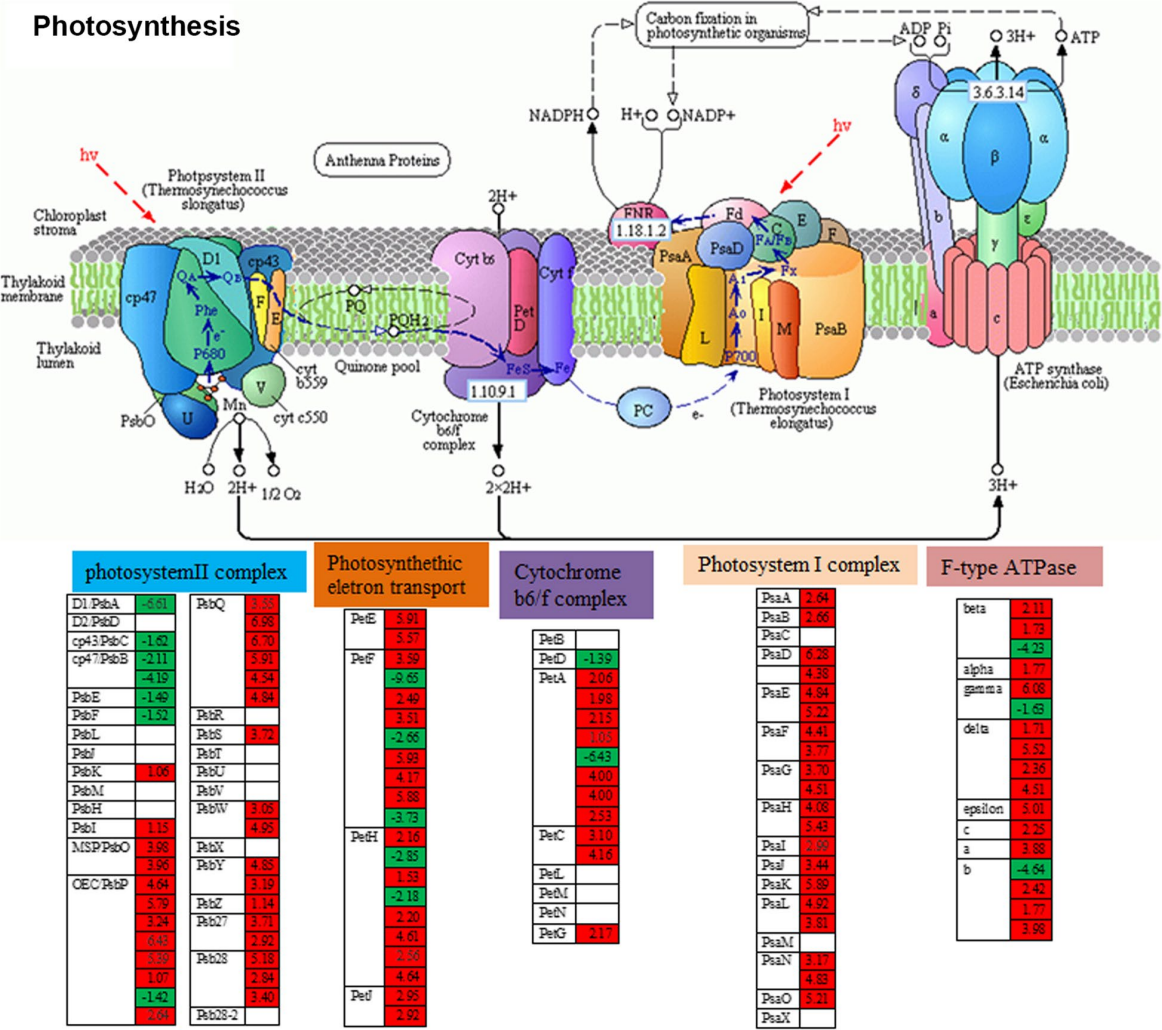
The DEGs involved in photosynthesis. Leaves from maize with added γ-PGA under drought stress were collected for RNA sequencing. Absolute values of log2 (CK+ γ-PGA/CK) ≥1 and FDR < 0.001 were used as the criteria for DEGs. The color of the box represents up-(red) and downregulated (green)- (CK+ γ-PGA/CK) genes, and the value in the box is the log2 (CK+ γ-PGA/CK) of the genes in the leaves (CK+ γ-PGA/CK) under drought stress. The photosynthesis pathway was identified via KEGG (http://www.genome.jp/kegg/)

As known, drought could significantly reduce the photosynthetic capability of plants. However, KEGG analysis showed that under drought stress, compared with the control plants, the photosynthesis related genes of maize treated with γ-PGA were significantly enriched (Fig. 3B), with most of the related genes being dramatically upregulated. As shown in Fig. 4 and Additional file 11: Table S1, most of the DEGs in the photosystem II complex were upregulated, except for all *PsbA*, *PsbB*, *PsbC*, *PsbE*, *PsbF* and 1 *PsbP*, which were downregulated. In the photosystem I complex, all of the DEGs were upregulated. In the cytochrome b6/f complex, 7 genes encoding PetA, 2 genes encoding PetC and 1 gene encoding PetG were upregulated, while only 1 gene encoding PetD and 1 gene encoding PetA were downregulated. In the photosynthetic electron transport, 16 genes encoding PetE, PetF, PetH and PetJ were all up-regulated except for 3 genes encoding PetF and 2 genes encoding PetH. In the F-type ATPase complex, except for 1 gene encoding beta, 1 for gamma and 1 for b which were downregulated, the other 14 genes encoding alpha, beta, gamma, delta, epsilon, a, b and c subunits respectively were upregulated. Additionally, all DEGs (67 genes) encoding antenna proteins were also upregulated (Additional file 4: Fig. S4). To confirm the results, 14 genes with different transcript abundances were validated by real-time RT–PCR (Additional file 5: Fig. S5). The expression of these genes showed good consistency between the two detection methods. Moreover, the motifs in the promoter regions of these genes were analyzed, and higher percentage of drought, low-temperature, salicylic stress and ABA response elements were found (Additional file 6: Fig. S6, Additional file 7: Fig. S7).

### γ-PGA promoted ABA accumulation and affected ABA signaling to improve drought resistance in maize

ABA, as an important drought response hormone, plays an important role in the response of maize to abiotic stress. Based on KEGG pathway analysis, DEGs related to the carotenoid biosynthesis pathway which contains the ABA biosynthesis pathway were significantly enriched (Fig. 3B), and γ-PGA could promote ABA accumulation under drought conditions (Fig. 1B). *CHY2*, *ABA1*, *NCED*, *ABA2* and *AAO3* were reported to be involved in ABA biosynthesis [35–38], and 8′-hydroxyase was reported to play an important role in the catabolism of ABA [39]. The RNA-seq results showed that 2 genes encoding CHY2, 7 genes encoding ABA1, 3 genes encoding NCED, 2 genes encoding ABA2, and 1 gene encoding AAO3 were significantly upregulated, while 2 genes encoding 8′-hydroxyase were downregulated. In addition, ABA signaling pathway related genes, including ABA receptor (PYR/PYL), PP2C, SnRK2 and ABFs were also differentially expressed. Among these DEGs, 3 for PYL, 4 for SnRK2, and 4 for ABF were upregulated, and 10 for PP2C were downregulated (Additional file 8: Fig. S8).

### γ-PGA affected the bacterial community diversity and structure of rhizospheric soil

**Fig. 5:**
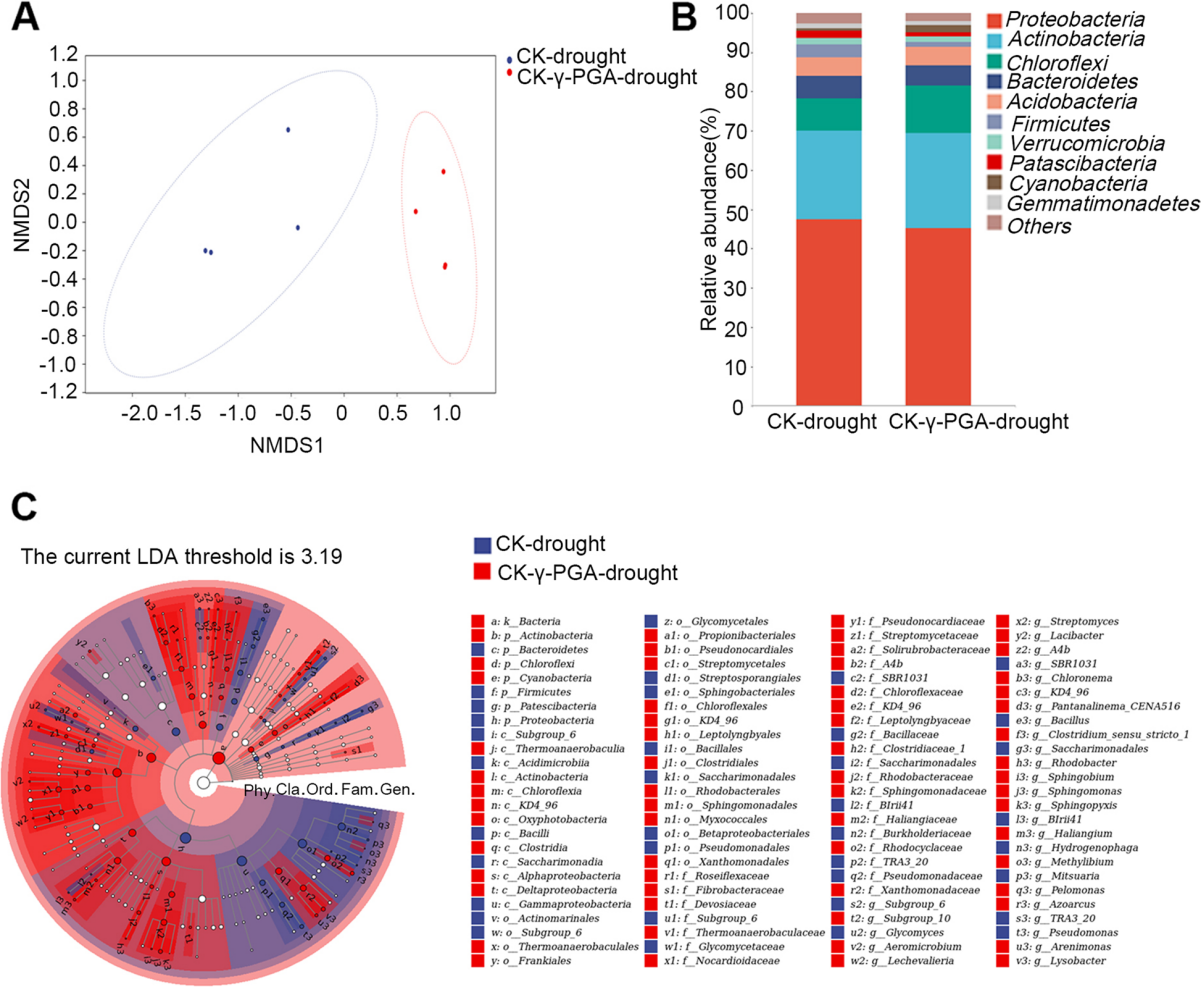
NMDS, relative abundance and LEfSe analysis. **A** Nonmetric multidimensional scaling (NMDS) shows the grouping patterns of the samples based on the weighted UniFrac distance of all community. Each colored dot represents a sample. **B** The influence of γ-PGA on the relative abundances of bacterial communities at the phylum level in the rhizosphere soil of maize. **c.** LEfSe analysis (LDA ≥ 3) shows the species with the most significant variation in the rhizosphere soil of control and γ-PGA supplemented maize under drought stress

In order to study the influence of γ-PGA on bacterial community diversity under drought stress, the relative abundance and diversity of maize rhizospheric soil bacteria were analyzed by high-throughput sequencing of 16S rRNA. The species curve showed that the samples were representative enough to obtain a true bacterial community (Additional file 9: Fig. S9). NMDS (stress = 0.00422) of the weighted UniFrac distance ordinations was conducted (Fig. 5A), and the results indicated that the bacterial community composition of the soil with γ-PGA application shifted compared with that of the soil without γ-PGA under the drought stress. The communities in maize rhizospheric soil with γ-PGA were grouped together and significantly separated from those in soil without γ-PGA under drought stress. The high-quality sequences that were obtained belonged to 36 phyla, among which the main phylum was *Proteobacteria*, followed by *Actinobacteria*, *Chloroflexi*, *Bacteroidetes*, and *Acidobacteria*. Although the diversity of the bacterial community changed after the addition of γ-PGA under drought stress, the predominant phyla were similar. There was no difference in species composition among these samples, but the relative abundances of some species changed (Fig. 5B). Compared to the control, the relative abundances of *Actinobacteria* and *Chloroflexi* were higher in soil supplemented with γ-PGA under drought stress. LEfSe analysis (LDA ≥ 3) showed the species with the most significant variation (Fig. 5C). Under drought stress, the application of γ-PGA could significantly enrich *Actinobacteria*, *Chloroflexi* and *Cyanobacteria* at the phylum level, while *Alphaproteobacteria* and *Deltaproteobacteria* were enriched at the class level. At the genus level, bacteria such as *Rhodobacter*, *Sphingobium*, *Sphingomonas*, *Sphingopyxis*, *Haliangium*, *Methylibium*, *Lysobacter*, *Azoarcus* and *Arenimonas* of *Proteobacteria*, *Aeromicrobium*, *Lechevalieria* and *Streptomyces* of *Actinobacteria*, *Subgroup_10* of *Acidobacteria*, *Clostridium* and *Pelotomaculum* of *Firmicutes*, *Chloronema*, *A4b* and *KD4–96* of *Chloroflexi* were dominant in γ-PGA supplemented rhizosphere soil under the persistent severe drought conditions. The abundances of these genera in maize rhizospheric soil with γ-PGA addition were all higher than those of the control (Additional file 10: Fig. S10), while *Bacillus* of *Proteobacteria* dominated in the control (Fig. 5C).

## Discussion

### Exogenous application of γ-PGA could significantly enhance the drought resistance of maize

Among all abiotic stresses, drought has the greatest impact on soil organisms and plants [40]. Drought could adversely affect the important physiological and biochemical processes of plants, resulting in serious crop yield losses worldwide [41]. It is critical to improve plant tolerance to drought stress. As a natural and environmentally friendly biopolymer, γ-PGA has been widely used in agricultural production [42]. However, there are few reports about the effect and mechanism of γ-PGA on the drought resistance of plants, especially crops. In this study, the effect of γ-PGA on maize drought resistance and its comprehensive mechanism were first reported by RNA-seq and rhizosphere soil bacterial community diversity analyses.

The effects of the exogenous application of γ-PGA on dry weight, and the contents of ABA, soluble sugar, proline and chlorophyll in maize leaves under severe drought stress were characterized. These physiological indices have often been used to evaluate the drought resistance of plants. As osmoprotectants, proline and soluble sugar can allow plants to make osmotic adjustments under drought stress [43]. Proline has a strong hydration ability, which can protect the cell structure and enzymes, reduce cell acidity and regulate redox potential under stress. ABA is considered to be the most critical hormone regulating tolerance to drought stress. Drought stress could trigger a large increase in ABA biosynthesis. As a key chemical messenger of drought signals, ABA can activate a series of signal transduction reactions to regulate stomatal closure, calcium signal and the expression of some ABA-responsive genes to resist the drought stress. Drought stress can significantly decrease the chlorophyll content of leaves [44, 45]. Plants with higher chlorophyll contents under drought stress could use light energy more efficiently and have better drought resistance. In this study, we found that under drought stress, γ-PGA could promote the accumulation of ABA, soluble sugar, proline and chlorophyll, and the drought resistance of maize was significantly enhanced by adding γ-PGA. In addition, γ-PGA could increase the dry weight of maize under drought stress, indicating that maize supplemented with γ-PGA could still maintain a certain level of growth compared with the control. To observe the root morphology under drought stress more directly, PEG6000 was used to simulate the drought treatment in the solution culture process. The results showed that, under PEG treatment, maize plants supplemented with γ-PGA had more developed roots than control plants, which could cause the plants to absorb deeper and more water from the soil during drought stress.

### The mechanism of enhanced drought resistance by exogenous application of γ-PGA at the molecular level

To explore the molecular mechanism of enhanced drought resistance by exogenous application of γ-PGA, the differentially expressed genes (DEGs) in the leaves were evaluated by RNA-seq analysis. KEGG analysis showed that photosynthesis related genes were significantly enriched which was consistent with the increase in the photosynthetic rate in the maize treated with γ-PGA under drought stress. Most of the photosynthesis-related genes, including 20 genes in photosystem I, 28 genes involved in photosystem II, 16 genes in photosynthetic electron transport, 10 genes in the cytochrome b6/f complex, 14 genes in the ATPase complex, and 31 genes encoding antenna proteins (9 genes encoding the LHCI complex and 22 genes encoding the LHCII complex), were dramatically upregulated in γ-PGA treated maize compared with the control. Photosynthesis is one of the main processes affected by drought [46]. However, under severe drought stress, the photosynthesis related genes in maize treated with γ-PGA still maintained a higher expression level than the control, which may be the main reason for the higher drought resistance of maize treated with γ-PGA, while the reduced chlorophyll contents under drought in the control led to the inactivation of photosynthesis. In this study, we also found that γ-PGA treatment improved the water holding capacity of maize roots. Therefore, even under drought conditions, the wilting of maize leaves was delayed than that of the control group, and the stomata may also be not completely closed, and some photosynthesis could be carried out. Therefore, the stomatal conductance and photosynthesis in the maize treated with the γ-PGA were significantly higher than that of the control group (Fig. 1B).

**Fig. 6:**
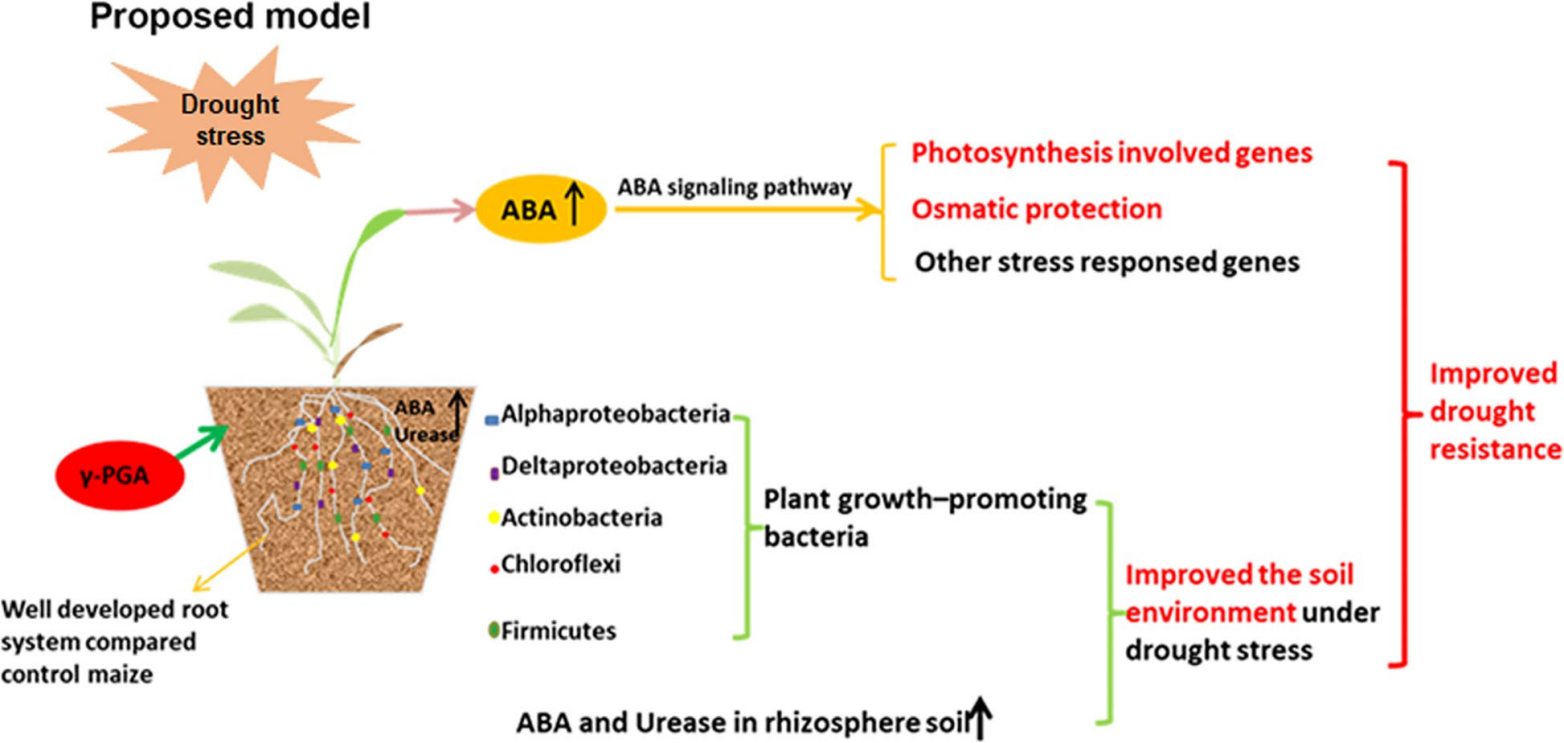
Proposed model for the role of γ-PGA in maize under long-term drought. γ-PGA can improve the drought resistance of maize by regulating the expression of ABA biosynthesis-related genes, ABA signal transduction-related genes, photosynthesis-related genes and other stress-responsive genes (osmotic protection, stress response and protein folding genes) and enriching plant-promoting bacteria such as *Actinobacteria*, *Chloroflexi, Firmicutes, Alphaproteobacteria* and *Deltaproteobacteria.* In addition, the ABA content and urease activity in maize rhizosphere soil also increased

ABA is considered to be the most critical hormone involved in the adaptive responses of plants to drought stress. DEGs related to the carotenoid biosynthesis pathway which contains the ABA biosynthesis pathway were also found to be significantly enriched in this study. In ABA biosynthesis, β-carotene is first converted to zeaxanthin by the CHY2 enzyme firstly, and the epoxidation of zeaxanthin and antheraxanthin to violaxanthin is subsequently catalysed by zeaxanthin epoxidase (ZEP/ABA1) [35]. Violaxanthin is converted to 9-cis-violaxanthin after a series of structural modifications. The next step is also a rate-limiting step, that is, 9-cis-violaxanthin is converted to xanthoxin under the catalysis of 9-cis-epoxycarotenoid dioxygenase (NCED) [36]. Subsequently, xanthoxin is converted to abscisic aldehyde, and then ABA is produced by two-step reaction via ABA-aldehyde. The enzyme (alcohol dehydrogenase/reductase) encoded by ABA2 catalyzes the first step of this reaction and generates ABA aldehyde [37], and abscisic aldehyde oxidase encoded by AAO3 catalyzes the last step of ABA synthesis [38]. In this study, it was found that ABA biosynthesis related genes including 2 genes encoding CHY2, 7 genes encoding ABA1, 3 genes encoding NCED, 2 genes encoding ABA2, and 1 gene encoding AAO3 were significantly upregulated, while 2 genes encoding 8′-hydroxyase which plays an important role in the catabolism of ABA were downregulated in maize with the application of γ-PGA. The expression level of these DEGs led to an increase in ABA levels in γ-PGA-treated maize under drought stress. The results indicated that γ-PGA could promote ABA accumulation under drought conditions, and the accumulation of ABA can activate the core ABA signaling pathway including the PYR/PYL/RCAR receptor, PP2C proteins, SnRK2 family members, AREB/ABF transcription factors and downstream regulatory genes, as well as the ABA-activated signaling pathway to resist drought stress [47]. In addition, many reports have shown that among the promoters of stress-responsive genes, there was a major cis-acting element (ABRE) which was regarded to be necessary for the ABA response [48]. We found that ABREs were present in the promoters of these upregulated photosynthesis related genes, suggesting that these genes may also be regulated by ABA. In addition, it was also found that many stress-responsive genes, including those in response to abiotic stimulus, were significantly enriched (Fig. 6).

### The effect of γ-PGA on the rhizosphere bacterial community diversity under drought stress

Many reports have shown that drought stress has a great impact on soil microbial communities and plays an important role in regulating plant response to drought stress [49]. Drought stress could lead to a significant reduction in microbial biomass [50–52] and change the composition of plant rhizosphere microbes. The drought tolerance of plants is related to changes in the relative abundance of specific bacterial groups [29–32, 40]. Although our understanding of the interaction between plants and soil microbes in drought responses is advancing, most of the knowledge comes from non-crop plants. The results of this study showed that the application of γ-PGA under drought stress did not affect the species of dominant bacteria, but changed the bacterial community diversity. Under drought stress, *Actinobacteria* and *Chloroflexi* were significantly enriched in soil supplemented with γ-PGA (Fig. 5C). *Actinobacteria* and *Chloroflexi* were reported to be the most prominent phyla under drought conditions [53]. *Actinobacteria* was previously found to promote the decomposition or formation of humus, making it easier to be absorbed [54, 55], and it was also reported to have an important role in plant defense and growth promotion [56–58]. In this study, it was also found that *Alphaproteobacteria* and *Deltaproteobacteria* were enriched at the class level after the addition of γ-PGA. Most members of *Proteobacteria* were reported to play important roles in nitrogen fixation [59, 60]. LEfSe analysis (LDA ≥ 3) shows that, at the genus level, *Sphingobium*, *Sphingomonas*, *Sphingopyxis*, and *Haliangium* of *Proteobacteria* were enriched in the rhizosphere soil of the maize treated with the γ-PGA under drought stress. And the *Actinobacteria* genus *Aeromicrobium*, *Lechevalieria* and *Streptomyces* were also enriched under the drought stress after the γ-PGA treatment. Among the three *Actinobacteria* genus, the genus *Streptomyces* was reported to enhance the drought resistance of the wheat seedling in water-stressed soils [61]. In addition, the genus *Subgroup_10* of *Acidobacteria*, *Clostridium* and *Pelotomaculum* of *Firmicutes*, which was previously reported to promote plant growth through nitrogen fixation, phosphate solubilization and production of plant hormones [54], were also found to be significantly enriched in the γ-PGA supplemented soil in this study.

### The possible mechanism of γ-PGA affecting the drought resistance of maize

It is worth noting that γ-PGA increased the urease activity of rhizosphere soils of maize under severe drought stress (Table 1). The activities of soil urease play an important role in soil nitrogen transformation, which produces NH_3,_ NH_4_^+^ and CO_3_^2−^ in the process of urea hydrolysis and provides nutrition for plants. The results implied that exogenous application of γ-PGA could contribute to improving the soil biochemical reaction and plant growth under the drought stress conditions. In addition, interestingly, we also detected a significant increase in the ABA content in the rhizosphere soil after γ-PGA application, which also plays an important role in the drought resistance of maize. The mechanism of the increase of urease activity and ABA content in soil by exogenous application of γ-PGA needs further study.

γ-PGA can improve the relative water content of the soil and the soil physicochemical properties under the drought conditions which could also affect microbial community of the soil. The research also showed that γ-PGA could also improve the development of the maize root system, and thus affecting the microbial community in the rhizosphere soil. Studies have shown that drought stress can change the amount and composition of root exudates [62, 63], which may lead to selective enrichment of rhizosphere microorganisms. And the root can counteract the hyperosmotic conditions created by drought through the accumulation of osmotic substances [64], which enable the plants to retain sufficient internal water to sustain its viability. In addition, γ-PGA may serve as a signal substance to induce the expression of a series of drought stress related genes in plants to improve drought resistance of maize [65], and in our study, the RNA-seq results also confirmed that many signaling and stress related genes had been differently expressed after γ-PGA treatment.

Our results showed that exogenous application of γ-PGA not only affected the physicochemical indices and gene expression related to drought resistance in plants but also profoundly affected the microbial community and physicochemical properties of rhizosphere soil (Fig. 6). In this study, we investigated the mechanism of γ-PGA in improving drought resistance of maize from the gene expression level and the changes of microbial community in rhizosphere root soil, which not only provided more information for further gene editing or transgenic technology to solve the problem of the drought resistance in plants, but also provide the guidance for improving the soil environment under drought conditions.

## Conclusions

Our study demonstrated that the exogenous application of γ-PGA could significantly enhance the drought resistance of maize under severe drought stress. γ-PGA can regulate the expression of ABA biosynthesis-related genes, ABA signal transduction related genes, photosynthesis-related genes and other stress-responsive genes. At the same time, γ-PGA could enrich the plant-promoting bacteria such as *Actinobacteria*, *Chloroflexi*, *Firmicutes*, *Alphaproteobacteria* and *Deltaproteobacteria*. This study highlighted the possibility of using γ-PGA to improve crop drought resistance and the soil environment under drought conditions.

## Materials and methods

### Plant materials and drought treatments

Maize (KN5585) seeds were sown in a soil box (10 cm*10 cm*10 cm). When seeds germinated, the seedlings were watered with different concentrations (0, 50, 70, and 100 mg/L) of γ-PGA (10 kDa) solution, and grown in greenhouse at 28 ± 2 °C under nature light, and 25 ± 2 °C at night. At the three-leaf stage, all seedlings were exposed to drought stress treatment by stopping watering to select the most suitable concentration of γ-PGA. After drought for 7 days (the soil water content decreased to 4.9%, and the control plants wilted seriously), the seedlings were rewatered, and the surviving rate after 7 d drought stress was calculated. After rewatering for 1 day, the recovered maize plants treated with γ-PGA were recorded and compared with the control plants. The 50 mg/L γ-PGA treatment was selected for the subsequent experiment according to the results of the drought lethality test. The physiological parameters including photosynthetic parameters (net photosynthetic CO_2_ assimilation rate, stomatal conductance) and contents of soluble sugar, proline, chlorophyll and ABA of the maize added with γ-PGA and the control were measured after 5 days of treatment (soil water content decreased to 9.8%). The soil water content was monitored by using soil moisture content meter (TZS, TOP instrument, China). Each experiment had at least three biological repetitions, and the determination of photosynthetic parameters was repeated at least five times. The leaves were collected for RNA sequencing. Finally, the dry weights of the plants under drought conditions were measured.

In the experiment using PEG to simulate drought stress, maize (inbred line KN5585) seeds were surface sterilized using 75% alcohol and germinated on moist filter paper in sterile petri dishes (diameter:12.5 cm) in the dark at 28 °C. After 4 days, the germinated seeds were transferred to culture flasks (height: 15 cm, diameter: 7 cm) with Hoagland solution, and grown at 28 °C/25 °C (16 h light/8 h dark) until the maize reached to the two-leaf stage. Then, the maize seedlings were divided into four groups and cultured as follows: Group 1, cultured with Hoagland solution only; Group 2, cultured with Hoagland solution supplemented with 18% (m/v) PEG6000 (− 0.77 MPa) solution; Group 3, cultured with Hoagland solution supplemented with γ-PGA (10 kDa, 50 mg L^− 1^); Group 4, cultured with Hoagland solution supplemented with γ-PGA (10 kDa, 50 mg L^− 1^) + 18% (m/v) PEG6000. The nutrient solution was renewed every 2 days, aerated with a mini air pump and supplemented with fresh solution. The phenotypes of the plants were examined, and fresh weights, the solute potential and the relative water content (RWC) were measured.

### Determination of physiological parameters

The leaf and root disks from the plants were excised, and the fresh weights (FWs) were recorded immediately. The dry weights (DWs) of the leaves were obtained after drying in an oven at 80 °C. The determination of relative water content (RWC) was based on the method of Smart [66], fresh leaves were weighed quickly to obtain fresh weight (FW), the leaves were then soaked in distilled water for 4 h to obtain the turgid weight (TW). The leaves were then dried at 80 °C, then the dry weight was measured. Finally, the RWC was calculated as follows: RWC (%) =100% × (FW–DW)/(TW–DW). The solute potential was calculated using the following equation: solute potential [MPa] = −concentration [mol/L] * gas constant [8.314 Pa*L/(mol*K)] * temperature [298.15 K]. The osmolyte concentrations (mol/L) were measured with an osmometer (Fiske Micro-Osmometer Model 210, USA) [67]. The photosynthetic parameters (net photosynthetic CO_2_ assimilation rate and stomatal conductance) were measured (at 28 °C, PAR:1000 μmol/m^2^/s) by a portable infrared gas analyser-based photosynthesis system (Yaxin-1105, China). The total soluble sugars in the leaves (approximately 100 mg) were extracted in boiling water for 30 min and determined by anthrone reagent using glucose as the standard according to the methods described by Yemm and Willis [68]. Proline was detected using the protocol described by Bates et al. [69]. Approximately 200 mg of the maize leaves was excised to measure the chlorophyll content following the method described by Arnon [70]. The ABA content was measured with an ELISA kit (code JM-01148P2, Jingmei Bio Inc., Jiangsu, China) according to the manufacturer’s protocol. The urease activity was determined according to the method described by Guan [71]. In this study, at least three biological repeats were sampled for one treatment, each replicate contained tissues from four plants, and the determination of photosynthetic parameters, RWC and the solute potential was repeated at least five times.

### RNA extraction and real-time RT–PCR

Total RNA was isolated from maize leaves following the manufacturer’s instructions using a HiPure RNA Kit (Magen, Guangzhou, China). 2 μg of total RNA was reverse transcribed into cDNA using reverse transcription kit (TAKARA). The cDNA was diluted to 200 μL with sterile DEPC water. Real-time RT–PCR of the candidate genes was performed by SYBR Green I Master Mix (Roche, Indianapolis, USA). Three biological and three technical replicates for each reaction were analyzed on a LightCycler 480 (Roche, USA) with a first step of 95 °C for 5 min followed by 40 cycles of 95 °C for 15 s, 60 °C for 15 s, and 72 °C for 15 s. Melting curves were generated using the following program: 95 °C for 15 s, 60 °C for 15 s, and for 15 s. *ZmTub* was used as an internal control. Data analysis was calculated by the 2^-ΔΔCT^ method. Significant differences between different samples were tested with IBM SPSS Statistics 22.0 software. Real-time PCR of the candidate genes and data analysis were performed and the primers used were listed in Additional file 12: Table S2.

### RNA sequencing and analysis

RNA sequencing and primary bioinformatics analysis were performed by BGI Tech Solutions Co., Ltd. (Shenzhen, China). Three biological replicates were performed for each treatment. Primary sequencing data (raw read) were produced by Illumina HiSeq™ 2000. After QC, raw reads were filtered into clean reads that were aligned to the reference sequences. The alignment data were utilized to calculate the distribution of reads on reference genes and the mapping ratio. Gene expression was measured as fragments per kilobase of transcript per million fragments mapped (FPKM) using Cufflinks. Differentially expressed genes (DEGs) were determined using DEseq2. The false discovery rate was used to adjust the *P* value. Genes with significant differences in expression, |log2Fold Change| ≥ 1, and adjusted *P*-value < 0.05 were considered as DEGs. GO analysis and pathway enrichment analysis of all DEGs (Q value≤0.05) were performed by AgriGO (http:// bioinfo.cau.edu.cn/agriGO/) and KEGG (http://www.genome.jp/kegg/). Promoter motif analysis was conducted using PlantCARE (http://bioinformatics.psb.ugent.be/webtools/plantcare/html/).

### Bacterial community analysis of maize rhizosphere soil

Two-leaf stage maize seedlings watered with γ-PGA (0 or 50 mg/L) were treated under drought stress and the soil moisture content was maintained at 8.0% by replenishment. After 30 days, the tightly bound soils of roots (serving as rhizosphere soils) were taken to analyze the microbial community, and three biological replicates were performed. Amplification and high-throughput sequencing of 16S rRNA from maize rhizosphere soil bacteria were performed as described by Wang et al. [72]. The primers of the V4 region of bacterial 16S rRNA were 338F (5′-ACTCCTACGGGAGGCAGCA-3′) and 806R (5′-GGACTACHVGGGTWTCTAAT-3′). High-throughput sequencing was conducted by an Illumina HiSeq 2000 (Illumina Inc., San Diego, USA). Nonmetric multidimensional scaling (NMDS) was performed on distance matrices and the coordinates were used to draw 2D graphical outputs. Taxa abundances at the phylum, class, order, family and genus levels were statistically compared among samples or groups by Metastats. LEfSe analysis (LDA ≥ 3) was carried out to obtain the important indicator taxa with significant changes in relative abundance.

### Statistical analysis

All data were from at least three biological replicates. The data were presented as the mean ± standard deviation (SD). The statistical analysis between the maize with and without γ-PGA treatment was performed using a T test and Duncan’s tests of one-way ANOVAs in SPSS (version 22.0.0.0). Significant differences were indicated by asterisks, *p < 0.05; **p < 0.01.

## Supplementary Information

**Additional File 1: Fig. S1.** Phenotypes of maize with added γ-PGA under drought stress. Phenotypes of maize with added different concentrations (0, 50 mg/L, 70 mg/L, 100 mg/L) of γ-PGA under the 7 d drought stress treatment and after re-watering for 1d. Bars=10 cm.

**Additional File 2: Fig. S2.** Determination of the RWC and the solute potential. (A) The solute potential of the maize with and without added γ-PGA under drought stress treatment with 18% PEG6000 solution. (B) The relative water content (RWC) of the maize with and without added γ-PGA under drought stress treatment with 18% PEG6000 solution. Values are means ± sd. Bars represent means ± sd (n=5 repeats). Significant differences are indicated by asterisks (**, P ≤0.01).

**Additional File 3: Fig. S3.** GO enrichment analysis of the DEGs. (A) GO enrichment analysis of the DEGs in leaves of maize with added γ-PGA and control maize. (B) The more detailed classification of the terms of response to abiotic stimulus.

**Additional File 4: Fig. S4.** The DEGs involved in photosynthesis-antenna proteins. The DEGs involved in photosynthesis. The leaf from the maize with added γ-PGA under drought stress was collected for RNA sequencing. The absolute values of log2 (CK+γ-PGA/CK) ≥1 and FDR < 0.001 were used as the criteria for DEGs. The color of the box represents up (red) and down (green)-regulated (CK+ γ-PGA/CK) genes, and the value in the box is the log2 (CK+ γ-PGA/CK) of the genes in the leaf (CK+ γ-PGA/CK) under drought stress. The pattern of photosynthesis-antenna proteins comes from KEGG (http://www.genome.jp/kegg/).

**Additional File 5: Fig. S5.** Validation of DEG identified in RNA-seq by real-time RT-PCR. The left heatmap showed the log2 fold changes of the DEGs identified in RNA-seq. The right bar graph was the results by real-time RT-PCR. Log2 values (CK-PGA-D/CK-D) were used to generate the plot. Expression levels of genes were analyzed by real-time RT-PCR, fold changes in transcripts were calculated by 2^-ΔΔCt^ method with *ZmTub* as an internal control.

**Additional File 6: Fig. S6.** The promoter elements analysis of the DEGs involved in photosynthesis. Different color squares represent different elements, and red squares represent ABA responsive elements.

**Additional File 7: Fig. S7.** The promoter elements analysis of the DEGs (coded by nuclear DNA) involved in photosynthesis-antenna proteins. Different color squares represent different elements, and red squares represent ABA responsive elements.

**Additional File 8: Fig. S8.** DEGs involved in ABA biosynthesis and ABA signaling pathway. Leaves from maize with added γ-PGA under drought stress was collected for RNA sequencing. The absolute values of log2 (CK+ γ-PGA/CK) ≥1 and FDR < 0.001 were used as the criteria for DEGs. The color of the box represents up (red) and down (green)-regulated (CK+ γ-PGA/CK) genes, and the value in the box is the log2 (CK+ γ-PGA/CK) of the genes in the leaf (CK+ γ-PGA/CK) under drought stress.

**Additional File 9: Fig. S9.** Species accumulation curves (boxplots) in rhizosphere soil of maize.

**Additional File 10: Fig. S10.** The relative abundance heatmap of the genus in rhizosphere soil of maize added γ-PGA identified by LEfSe analysis.

**Additional File 11: Table S1.** The overall of RNA-seq in this paper.

**Additional File 12: Table S2.** Primer sequence used in this paper.

**Additional File 13: Table S3.** DEGs involved in photosynthesis, photosynthesis-antenna protein, ABA biosynthesis and ABA signaling pathway.

## Data Availability

All datasets generated for this study are included in the article/Supplementary Materials. The RNA-Seq raw data have been uploaded to a public database: 10.6084/m9.figshare.14495775.v1 The data of 16 s rRNA from maize rhizosphere soil bacterial were deposited in the figshare database: 10.6084/m9.figshare.14496006.v1
