# Supplementary material for: Poly-γ-glutamic acid enhanced the drought resistance of maize by improving photosynthesis and affecting the rhizosphere microbial community
Source: BMC Plant Biol. 2022 Jan 3;22:11. doi: 10.1186/s12870-021-03392-w (PMC8722152; doi:10.1186/s12870-021-03392-w)
Supplement: Supplementary file 1 — Additional File 1: Fig. S1. Phenotypes of maize with added γ-PGA under drought stress. Phenotypes of maize with added different concentrations (0, 50 mg/L, 70 mg/L, 100 mg/L) of γ-PGA under the 7 d drought stress treatment and after re-watering for 1d. Bars=10 cm. [file 12870_2021_3392_MOESM1_ESM.docx]

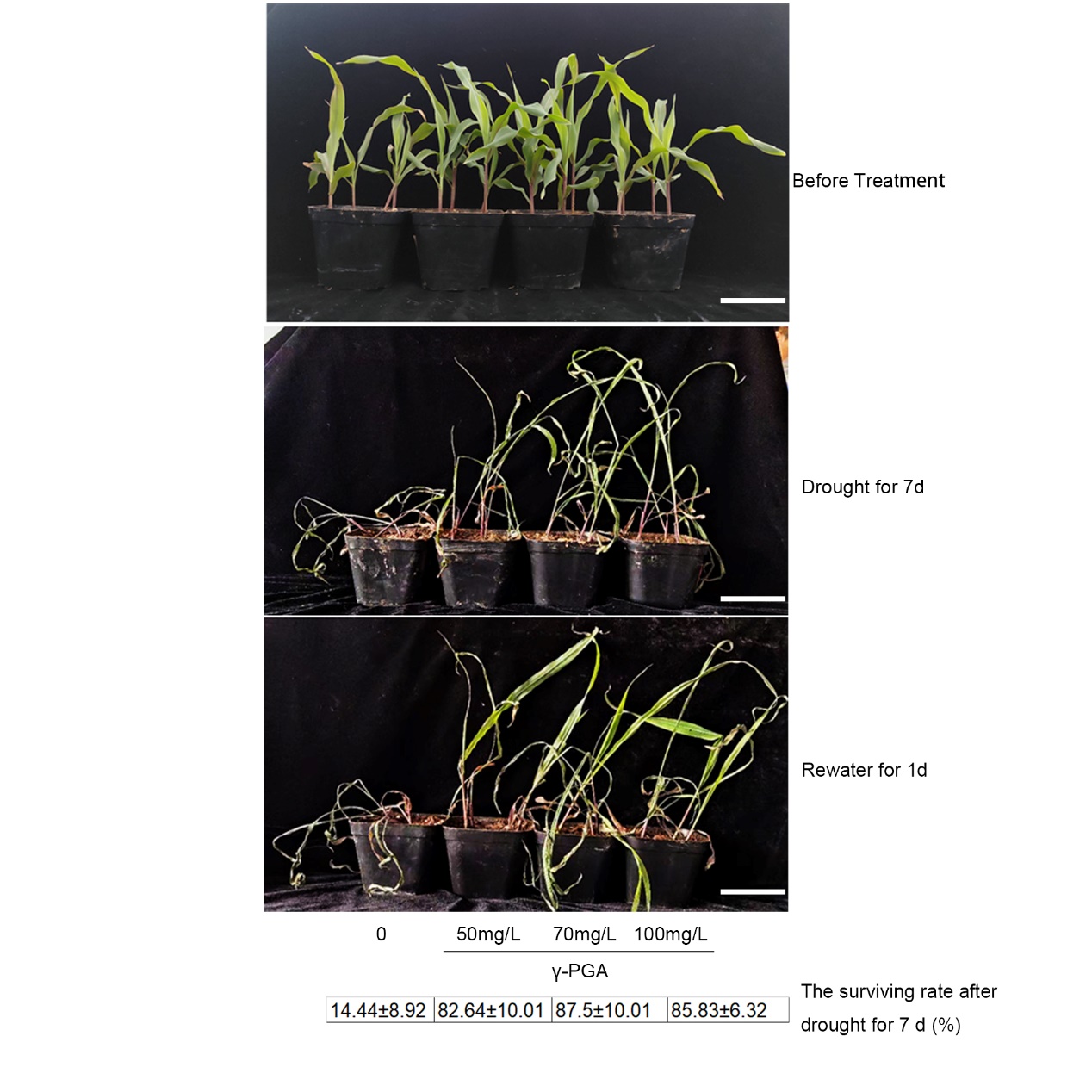


**Fig. S1** Phenotypes of maize with added γ-PGA under drought stress. Phenotypes of maize with added different concentrations (0, 50 mg/L, 70 mg/L, 100 mg/L) of γ-PGA under the 7 d drought stress treatment and after re-watering for 1 d. Bars=10 cm.
