# Supplementary material for: Poly-γ-glutamic acid enhanced the drought resistance of maize by improving photosynthesis and affecting the rhizosphere microbial community
Source: BMC Plant Biol. 2022 Jan 3;22:11. doi: 10.1186/s12870-021-03392-w (PMC8722152; doi:10.1186/s12870-021-03392-w)
Supplement: Supplementary file 2 — Additional File 2: Fig. S2. Determination of the RWC and the solute potential. (A) The solute potential of the maize with and without added γ-PGA under drought stress treatment with 18% PEG6000 solution. (B) The relative water content (RWC) of the maize with and without added γ-PGA under drought stress treatment with 18% PEG6000 solution. Values are means ± sd. Bars represent means ± sd (n=5 repeats). Significant differences are indicated by asterisks (**, P ≤0.01). [file 12870_2021_3392_MOESM2_ESM.docx]

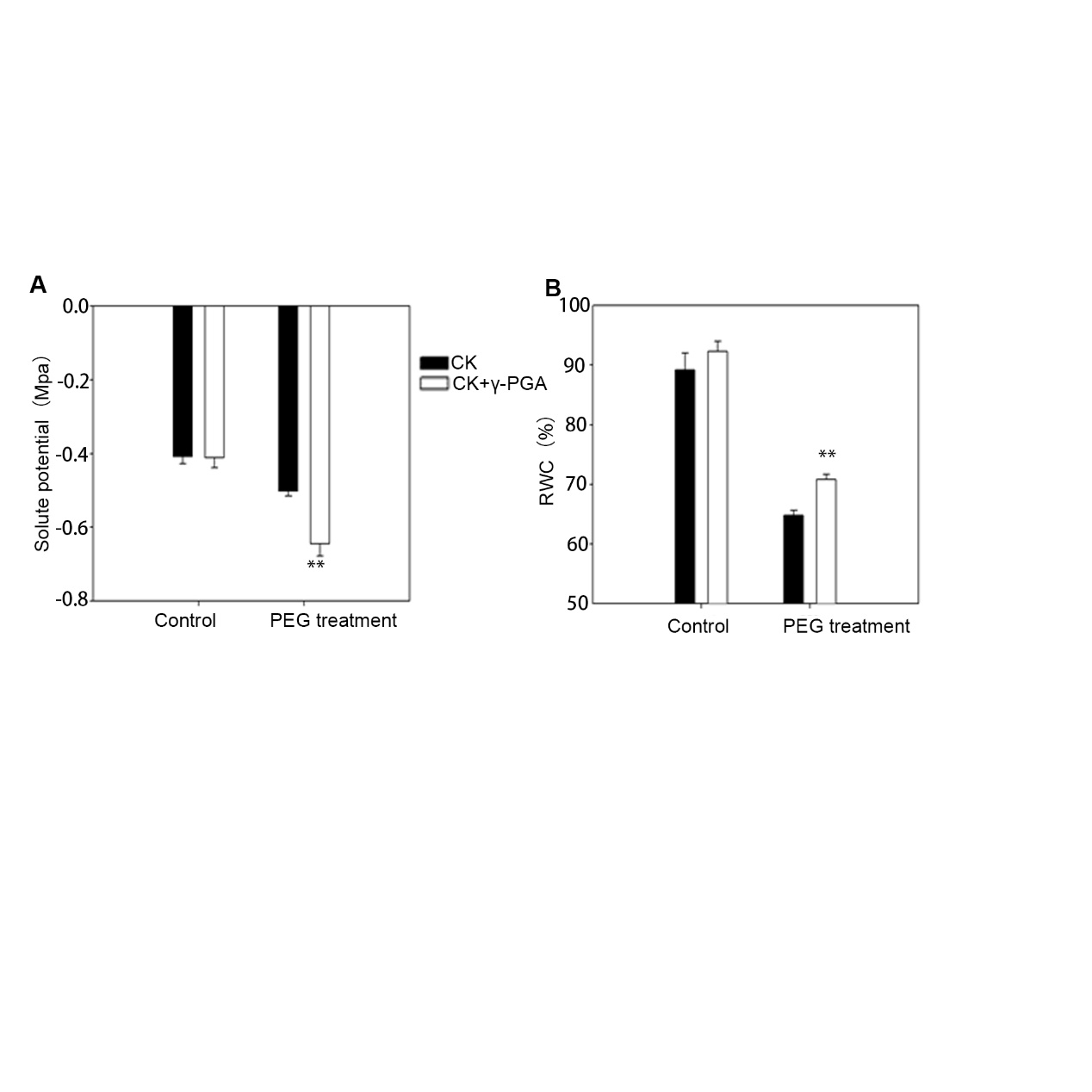


**Fig. S2** Determination of the RWC and the solute potential. **(A)** The solute potential of the maize with and without added γ-PGA under drought stress treatment with 18% PEG6000 solution. **(B)** The relative water content (RWC) of the maize with and without added γ-PGA under drought stress treatment with 18% PEG6000 solution. Values are means ± sd. Bars represent means ± sd (n=5 repeats). Significant differences are indicated by asterisks (**, P ≤0.01).
