# Supplementary material for: Poly-γ-glutamic acid enhanced the drought resistance of maize by improving photosynthesis and affecting the rhizosphere microbial community
Source: BMC Plant Biol. 2022 Jan 3;22:11. doi: 10.1186/s12870-021-03392-w (PMC8722152; doi:10.1186/s12870-021-03392-w)
Supplement: Supplementary file 3 — Additional File 3: Fig. S3. GO enrichment analysis of the DEGs. (A) GO enrichment analysis of the DEGs in leaves of maize with added γ-PGA and control maize. (B) The more detailed classification of the terms of response to abiotic stimulus. [file 12870_2021_3392_MOESM3_ESM.docx]

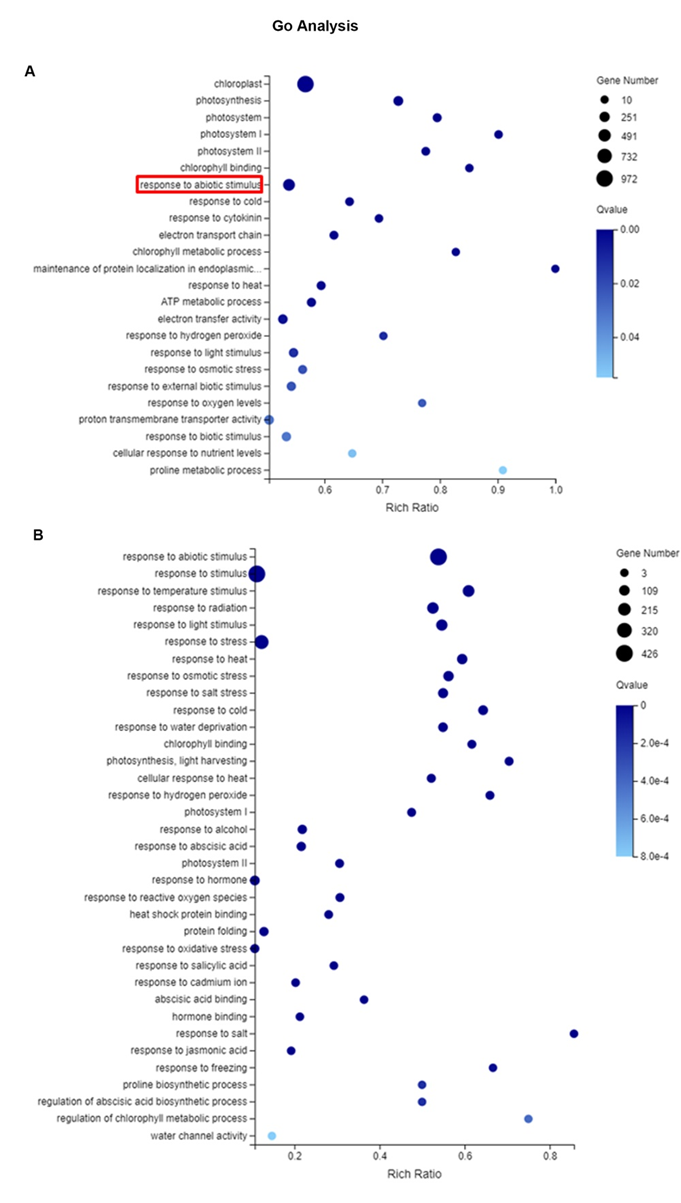


**Fig. S3** GO enrichment analysis of the DEGs. **(A)** GO enrichment analysis of the DEGs in leaves of maize with added γ-PGA and control maize. **(B)** The more detailed classification of the terms of response to abiotic stimulus.
