# Supplementary material for: Poly-γ-glutamic acid enhanced the drought resistance of maize by improving photosynthesis and affecting the rhizosphere microbial community
Source: BMC Plant Biol. 2022 Jan 3;22:11. doi: 10.1186/s12870-021-03392-w (PMC8722152; doi:10.1186/s12870-021-03392-w)
Supplement: Supplementary file 5 — Additional File 5: Fig. S5. Validation of DEG identified in RNA-seq by real-time RT-PCR. The left heatmap showed the log2 fold changes of the DEGs identified in RNA-seq. The right bar graph was the results by real-time RT-PCR. Log2 values (CK-PGA-D/CK-D) were used to generate the plot. Expression levels of genes were analyzed by real-time RT-PCR, fold changes in transcripts were calculated by 2-ΔΔCt method with ZmTub as an internal control. [file 12870_2021_3392_MOESM5_ESM.docx]

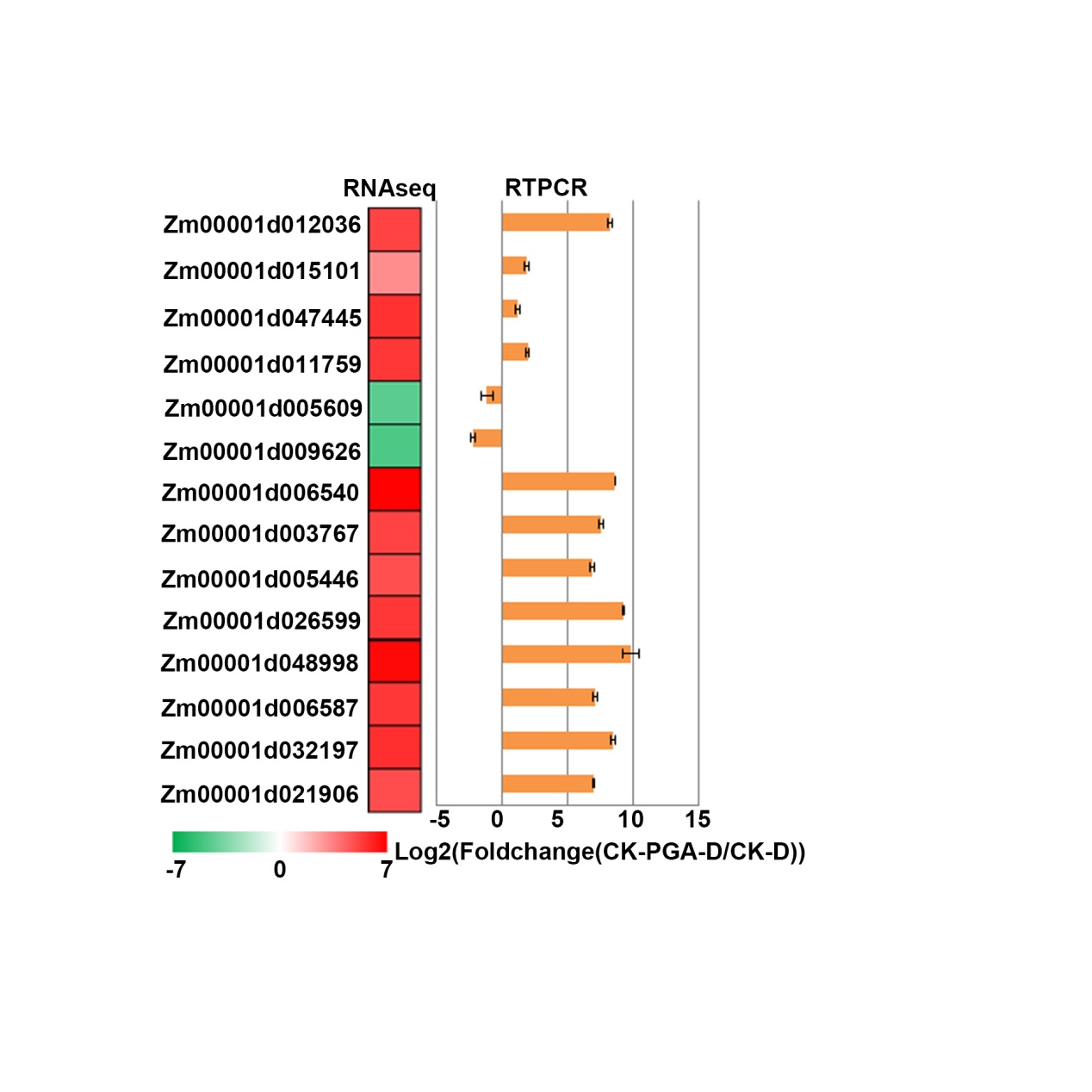


**Fig. S5** Validation of DEG identified in RNAseq by real-time RT-PCR. The left heatmap showed the log2 fold changes of the DEGs identified in RNAseq. The right bar graph was the results by real-time RT-PCR. Log2 values (CK-PGA-D/CK-D) were used to generate the plot. Expression levels of genes were analyzed by real-time RT-PCR, fold changes in transcripts were calculated by 2^-ΔΔCt^ method with *ZmTub* as an internal control.
