# Supplementary material for: Poly-γ-glutamic acid enhanced the drought resistance of maize by improving photosynthesis and affecting the rhizosphere microbial community
Source: BMC Plant Biol. 2022 Jan 3;22:11. doi: 10.1186/s12870-021-03392-w (PMC8722152; doi:10.1186/s12870-021-03392-w)
Supplement: Supplementary file 6 — Additional File 6: Fig. S6. The promoter elements analysis of the DEGs involved in photosynthesis. Different color squares represent different elements, and red squares represent ABA responsive elements. [file 12870_2021_3392_MOESM6_ESM.docx]

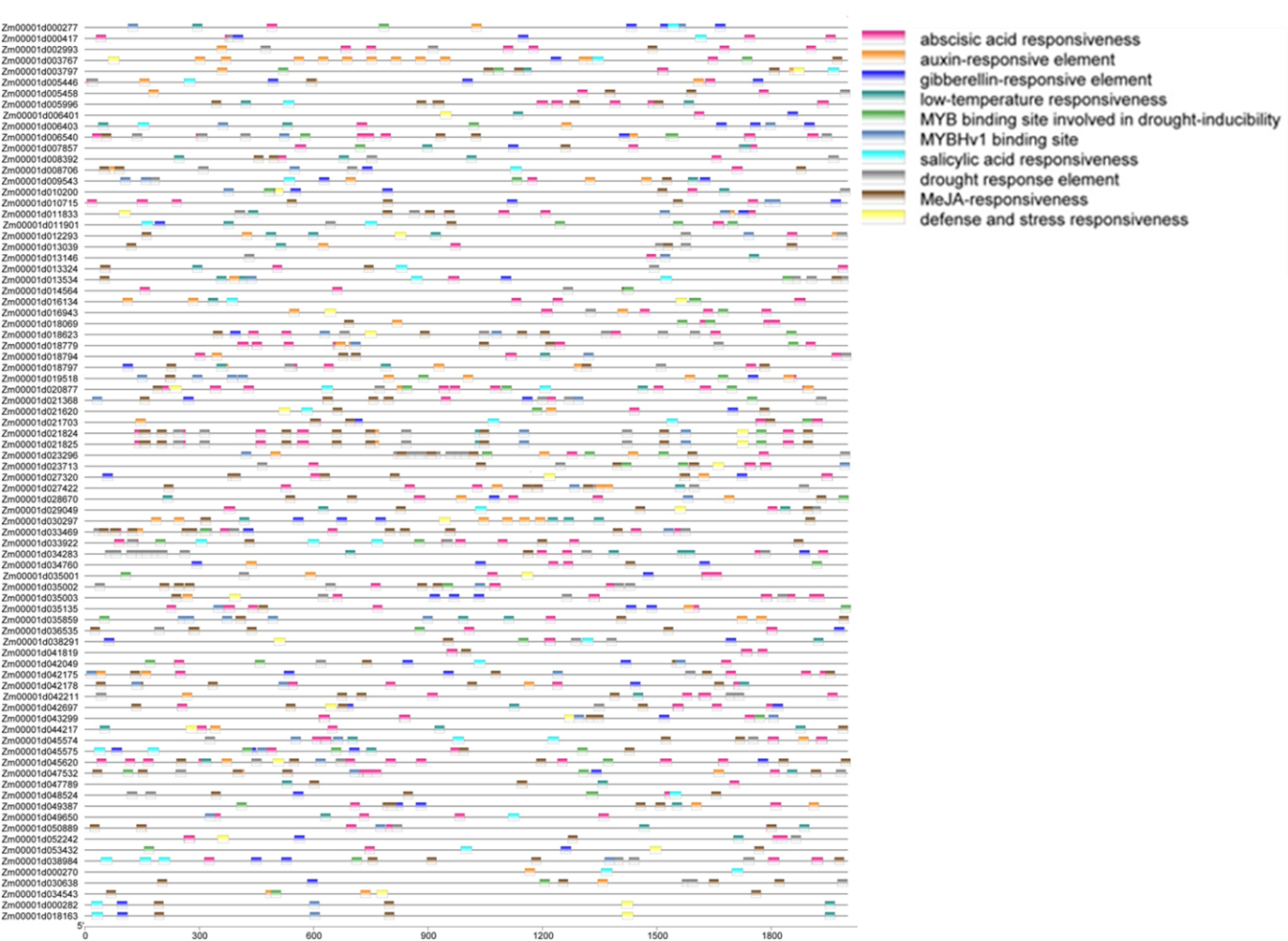


**Fig. S6** The promoter elements analysis of the DEGs involved in photosynthesis. Different color squares represent different elements, and red squares represent ABA responsive elements.
