# Supplementary material for: Poly-γ-glutamic acid enhanced the drought resistance of maize by improving photosynthesis and affecting the rhizosphere microbial community
Source: BMC Plant Biol. 2022 Jan 3;22:11. doi: 10.1186/s12870-021-03392-w (PMC8722152; doi:10.1186/s12870-021-03392-w)
Supplement: Supplementary file 8 — Additional File 8: Fig. S8. DEGs involved in ABA biosynthesis and ABA signaling pathway. Leaves from maize with added γ-PGA under drought stress was collected for RNA sequencing. The absolute values of log2 (CK+ γ-PGA/CK) ≥1 and FDR < 0.001 were used as the criteria for DEGs. The color of the box represents up (red) and down (green)-regulated (CK+ γ-PGA/CK) genes, and the value in the box is the log2 (CK+ γ-PGA/CK) of the genes in the leaf (CK+ γ-PGA/CK) under drought stress. [file 12870_2021_3392_MOESM8_ESM.docx]

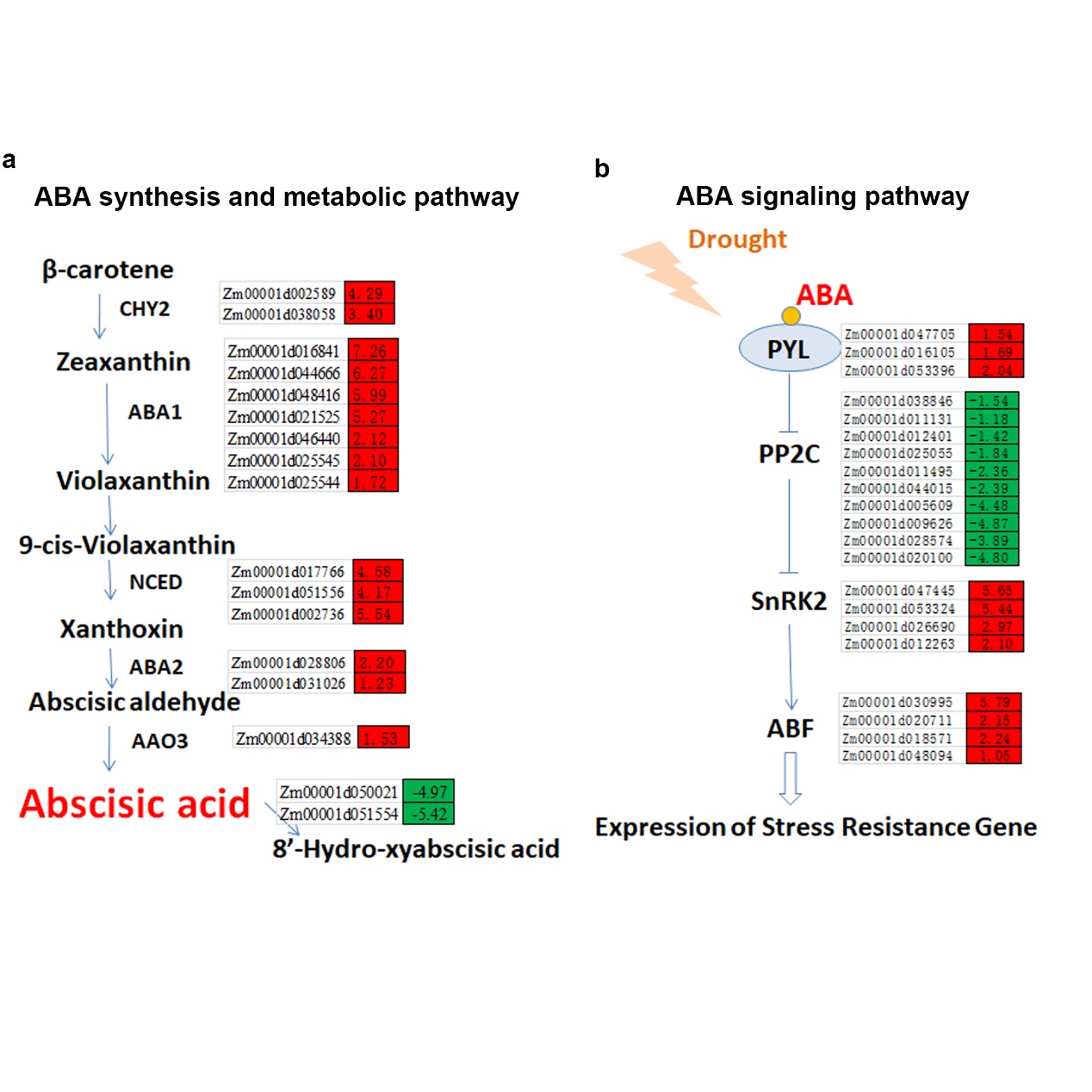


**Fig. S8** DEGs involved in ABA biosynthesis and ABA signaling pathway. Leaves from maize with added γ-PGA under drought stress was collected for RNA sequencing. The absolute values of log2 (CK+ γ-PGA/CK) ≥1 and FDR < 0.001 were used as the criteria for DEGs. The color of the box represents up (red) and down (green)-regulated (CK+ γ-PGA/CK) genes, and the value in the box is the log2 (CK+ γ-PGA/CK) of the genes in the leaf (CK+ γ-PGA/CK) under drought stress.
