# Supplementary figures and images for: Poly-γ-glutamic acid enhanced the drought resistance of maize by improving photosynthesis and affecting the rhizosphere microbial community
Source: BMC Plant Biol. 2022 Jan 3;22:11. doi: 10.1186/s12870-021-03392-w (PMC8722152; doi:10.1186/s12870-021-03392-w)

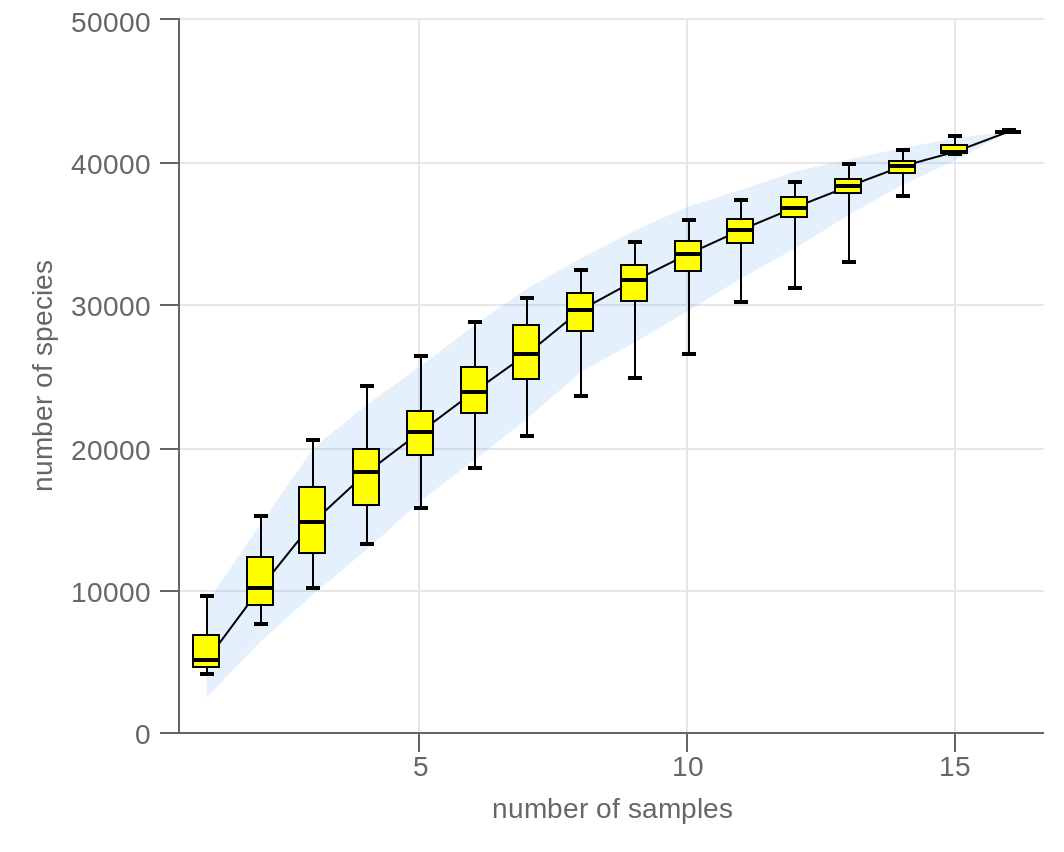


**Fig. S9** Species accumulation curves (boxplots) in rhizosphere soil of maize.

Supplement: Supplementary file 9 — Additional File 9: Fig. S9. Species accumulation curves (boxplots) in rhizosphere soil of maize. [file 12870_2021_3392_MOESM9_ESM.docx]
