# Supplementary material for: Poly-γ-glutamic acid enhanced the drought resistance of maize by improving photosynthesis and affecting the rhizosphere microbial community
Source: BMC Plant Biol. 2022 Jan 3;22:11. doi: 10.1186/s12870-021-03392-w (PMC8722152; doi:10.1186/s12870-021-03392-w)
Supplement: Supplementary file 10 — Additional File 10: Fig. S10. The relative abundance heatmap of the genus in rhizosphere soil of maize added γ-PGA identified by LEfSe analysis. [file 12870_2021_3392_MOESM10_ESM.docx]

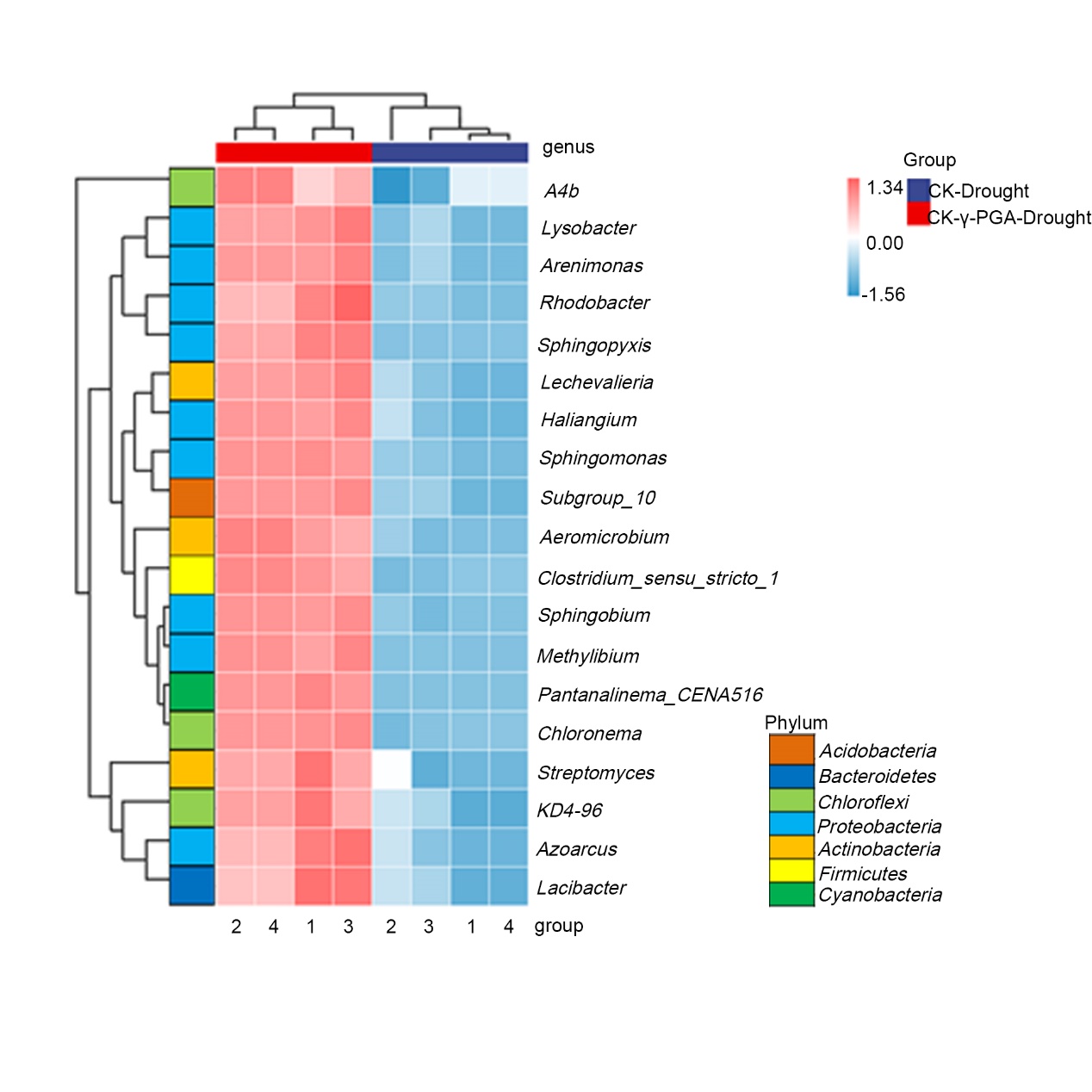


**Fig. S10** The relative abundance heatmap of the genus in rhizosphere soil of maize added γ-PGA identified by LEfSe analysis.
