# Supplementary material for: Poly-γ-glutamic acid enhanced the drought resistance of maize by improving photosynthesis and affecting the rhizosphere microbial community
Source: BMC Plant Biol. 2022 Jan 3;22:11. doi: 10.1186/s12870-021-03392-w (PMC8722152; doi:10.1186/s12870-021-03392-w)
Supplement: Supplementary file 11 — Additional File 11: Table S1. The overall of RNA-seq in this paper. [file 12870_2021_3392_MOESM11_ESM.docx]

Table S1. The overall of RNAseq in this paper

| Sample | Total Raw Reads (M) | Total Clean Reads (M) | Clean Reads Ratio(%) | Genome Mapping(%) | Gene mapping(%) | Uniquely Mapping(%) |
| --- | --- | --- | --- | --- | --- | --- |
| CK_D_1 | 26.1 | 22.14 | 84.82 | 88.3 | 64.81 | 72.29 |
| CK_D_2 | 23.92 | 21.94 | 91.73 | 88.23 | 65.18 | 72.48 |
| CK_D_3 | 23.92 | 22.53 | 94.17 | 88.39 | 65.49 | 72.46 |
| T_D_1 | 21.75 | 21.18 | 97.37 | 89.58 | 65.29 | 73.13 |
| T_D_2 | 21.75 | 21.24 | 97.65 | 89.51 | 65.45 | 72.98 |
| T_D_3 | 21.75 | 21.54 | 99.02 | 89.72 | 66.33 | 73.21 |
